# Dear Sir

**Published:** 2009-10

**Authors:** Mak Omar, Gerald W Friedland

**Affiliations:** Department of Endocrinology, Nelson R Mandela School of Medicine, University of KwaZulu-Natal, Durban

## Dear Sir

In the editorial by G Friedland,^1^ ‘Discovery of the function of the heart and circulation of blood’ he omits the important contribution made by Ibn al-Nafis (born 1213) on the subject.^2^,^3^ About 400 years before Harvey’s observation, Al-Nafis, a Syrian Arab physician correctly described the pulmonary circulation. In fact, Servitus, the Spanish physician (born 1511) whom he cites, was probably influenced by Al-Nafis in this regard.^2^

Of interest is that Al-Nafis was also the first to describe the constitution of the lungs and the interaction between the airways and blood vessels. He described what he termed small ‘pores’ between the pulmonary artery and vein (capillaries). In addition, he described the coronary circulation and rightly surmised that it was responsible for feeding the cardiac muscle.

## Reply

Dear Sir

I searched the internet and discovered that Dr Omar is correct.

Ibn al-Nafis did describe the pulmonary circulation.
